# Serum Levels of Glutamatergic (GRIN1) and Purinergic (P2RX1/P2RY2) Receptors in Patients with Fibromyalgia

**DOI:** 10.3390/ijms27073164

**Published:** 2026-03-31

**Authors:** Sevil Ceyhan Dogan, Gülcihan Cinar Kaya, Zuhal Tuncbilek, Mert Atas, Ayca Tas

**Affiliations:** 1 Department of Physical Medicine and Rehabilitation, Faculty of Medicine, Sivas Cumhuriyet University, Sivas 58140, Türkiye; drsevilceyhan@gmail.com (S.C.D.); mertatas.18@gmail.com (M.A.); 2 Department of Medical Services and Techniques, Yıldızeli Vocational School, Medical Laboratory Techniques Program, Sivas Cumhuriyet University, Sivas 58140, Türkiye; gulcihancinarkaya@cumhuriyet.edu.tr; 3 Department of Chemistry and Chemical Processing Technologies Services, Yıldızeli Vocational School, Biochemistry Program, Sivas Cumhuriyet University, Sivas 58140, Türkiye; zuhaltuncbilek@cumhuriyet.edu.tr; 4 Department of Medical Biochemistry, Faculty of Medicine, Sivas Cumhuriyet University, Sivas 58140, Türkiye

**Keywords:** fibromyalgia, GRIN1, P2RX1, P2RY2

## Abstract

Fibromyalgia (FM) is a chronic pain syndrome characterized by central sensitization, in which glutamatergic and purinergic signaling pathways are thought to play critical roles. This study aimed to evaluate the diagnostic potential of serum glutamate ionotropic receptor N-methyl-D-aspartate type subunit 1 (GRIN1), purinergic receptor P2X 1 (P2RX1), and purinergic receptor P2Y 2 (P2RY2) levels in patients with FM. A total of 93 newly diagnosed FM patients and 93 age- and sex-matched healthy controls were included in the study. Serum levels of GRIN1, P2RX1, and P2RY2 were measured using enzyme-linked immunosorbent assay (ELISA). Receiver operating characteristic (ROC) curve analysis was performed to assess the diagnostic performance of these biomarkers. ROC analysis demonstrated good diagnostic accuracy for all three biomarkers. The area under the curve (AUC) values were 0.817 for GRIN1, 0.778 for P2RX1, and 0.842 for P2RY2 (*p* < 0.001 for all). At optimal cut-off values, GRIN1, P2RX1, and P2RY2 showed sensitivities of 91.4%, 78.5%, and 92.5%, and specificities of 72.00%, 75.3%, and 80.6%, respectively. Serum GRIN1, P2RX1, and P2RY2 levels exhibit strong diagnostic performance in FM and may serve as promising biomarkers reflecting altered glutamatergic and purinergic signaling in disease pathophysiology.

## 1. Introduction

Fibromyalgia (FM) is a syndrome characterized by fatigue, chronic widespread pain, sleep disturbances, and decreased cognitive function, the etiology of which has not yet been fully elucidated [1]. Its worldwide prevalence varies between 2% and 4%, and it is more prevalent in women than in men [2]. FM is associated with neuroinflammation, neurotransmitter imbalance, and impairments in central sensory functions [3]. Hypersensitivity, particularly in pain processing mechanisms in the central nervous system, underlies both the exaggerated perception of signals affecting the peripheral nervous system and the spontaneous experience of pain. Therefore, FM is not only considered a musculoskeletal disease but also a central pain syndrome in which neurobiological processes are profoundly affected [4].

Recent studies have demonstrated that interactions between the purinergic, glutamatergic, and neuroimmune systems are important criteria in the pathophysiology of FM. In this context, specific receptors such as glutamate ionotropic receptor N-methyl-D-aspartate type subunit 1 (GRIN1), purinergic receptor P2X 1 (P2RX1), and purinergic receptor P2Y 2 (P2RY2)are gaining increasing importance due to their critical roles in processes such as increasing pain sensitivity through ion channels and regulating inflammatory responses. GRIN1 is an essential subunit of the N-methyl-D-aspartate (NMDA) receptor complex and plays a central role in neuronal communication, synaptic plasticity, and the transmission of pain signals [5]. Excessive activation of the NMDA receptor may lead to an exacerbation of FM symptoms by increasing neuronal hyperexcitability, which forms the molecular basis of central signaling [6].

It is well established in the literature that P2X7 and P2X4 receptors have been more extensively studied in fibromyalgia-related neuroinflammation and pain mechanisms [7,8]. However, the P2RX1 receptor has attracted attention as a potentially important but underexplored target in the context of fibromyalgia, due to its role in ATP-mediated signaling, vascular smooth muscle function, and peripheral biological processes [9,10]. In addition, purinergic receptors play an important role in the neurobiological process of FM. P2XR1 is a ligand-gated ion channel activated by ATP and is expressed in the dorsal root ganglia, peripheral nerve endings, and various regions of the spinal cord [11]. Increases in ATP during cellular stress, inflammation, or tissue damage lead to the generation of strong nociceptive signals through P2X receptors. Therefore, an increase in P2XR1 activity may strengthen the molecular basis of chronic pain susceptibility in addition to peripheral sensitization [12]. P2RX1 is a ligand-gated ion channel activated by ATP and is predominantly expressed in smooth muscle cells and platelets, where it contributes to the regulation of cellular responses [9]. Increased extracellular ATP levels under conditions such as inflammation and tissue damage trigger strong nociceptive signals via purinergic receptors [13]. Therefore, P2X receptors are generally considered key components of chronic pain mechanisms. In contrast, the circulating levels and clinical relevance of P2RX1 in fibromyalgia remain unclear. This gap highlights the scientific relevance of evaluating P2RX1 as a novel biomarker candidate.

P2YR2 is a member of the G protein-coupled purinergic receptor family and is activated by nucleotides such as ATP and UTP [14]. P2YR2 plays a critical role in tissue repair, inflammation, immune response, and neurotransmission processes by regulating intracellular calcium responses. The impairment of neuron–glia communication in FM, increased peripheral inflammatory responses, and abnormalities in sensory neuron function demonstrate the potential role of P2YR2 in this disease [13]. In contrast, P2RY2 is a member of the G protein-coupled purinergic receptor family and is activated by nucleotides, such as ATP and UTP. It plays a critical role in regulating intracellular calcium signaling and is involved in processes such as inflammation, immune responses, and tissue repair [9]. Increased inflammatory responses and disruptions in neuron–glia interactions further support the potential involvement of P2RY2 in chronic pain conditions [15].

The present study aimed to investigate serum levels of glutamatergic (GRIN1) and purinergic (P2RX1 and P2RY2) receptors in patients with fibromyalgia and evaluate their potential as novel biomarkers reflecting alterations in neuroinflammatory and pain-related signaling pathways. In addition, this study sought to explore the combined contribution of glutamatergic and purinergic systems to the pathophysiology of fibromyalgia by assessing the diagnostic performance and clinical relevance of these circulating proteins. By addressing the limited clinical evidence regarding P2RX1 and its role in fibromyalgia, this study aims to provide new insights into the systemic molecular mechanisms underlying the disease and identify potential targets for future diagnostic and therapeutic strategies.

## 2. Results

### 2.1. Demographic and Clinical Characteristics

When comparing the two groups in terms of age, the patient group was 44.66 ± 10.43 years old, and the control group was 42.84 ± 6.98 years old. The difference between the groups in terms of age was insignificant (*p* > 0.05). Individuals in the patient group were 2.2% male and 978% female, while in the control group, 1.1% were male and 98.9% were female. There was no difference between the groups in terms of gender (*p* > 0.05). When individuals in both groups were compared in terms of sleep disturbance, fatigue, headache, morning fatigue, dry mouth, leg numbness, dry eyes, difficulty concentrating, feeling of swelling in soft tissues, and family history of FMS, the difference between the groups was found to be significant (*p* < 0.05). When compared in terms of occupation, it was insignificant (Table 1).

Patients are 4.78 times more likely to experience morning fatigue than healthy individuals, and this odds value is statistically significant. Patients are 2.56 times more likely to experience dry mouth than healthy individuals, and this odds value is statistically significant. Patients are 3.80 times more likely to experience leg numbness than healthy individuals, and this odds value is statistically significant. Patients are 5.06 times more likely to experience difficulty concentrating than healthy individuals, and this odds value is statistically significant. Patients are 2.45 times more likely to experience a feeling of swollen soft tissues than healthy individuals, and this odds value is statistically significant.

Table 2 presents the clinical characteristics associated with fibromyalgia as identified by multivariate logistic regression analysis. Headache, morning fatigue, dry mouth, leg numbness, difficulty concentrating, and soft tissue swelling sensation were found to be significant predictors of fibromyalgia. All variables included in the final model showed statistically significant odds ratios, indicating an increased likelihood of fibromyalgia in individuals presenting these clinical features.

### 2.2. Serum GRIN1, P2RX1, and P2RY2 Protein Levels

Within the scope of the study, serum GRIN1, P2RX1, and P2RY2 protein levels were measured in 93 patients with FM and 93 healthy individuals using the ELISA method. When individuals in both groups were compared in terms of serum GRIN1, P2RX1, and P2RY2 protein levels, the difference between the groups was found to be significant. Serum GRIN1, P2RX1, and P2RY2 protein levels in the patient group were higher than in the control group. Correlations of r = 0.61 were found between GRIN1 and P2RX1, r = 0.70 between GRIN1 and P2RY2, and r = 0.63 between P2RX1 and P2RY2. These correlations were statistically significant and of moderate strength (Table 3).

### 2.3. Diagnostic Performance of GRIN1, P2RX1, and P2RY2

**Figure 1:**
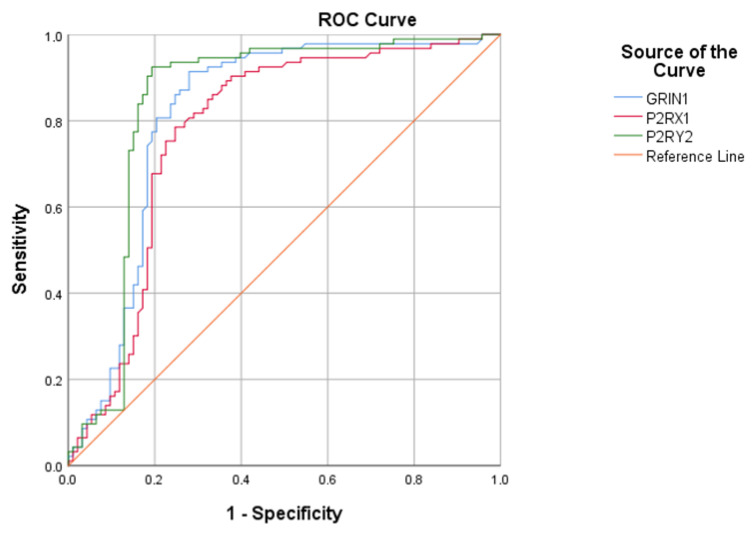
ROC curves of GRIN1, P2RX1 and P2RY2 proteins.

Receiver operating characteristic (ROC) curve analysis demonstrated good diagnostic performance for GRIN1, P2RX1, and P2RY2 in FM. The AUC values were 0.817 (95% CI: 0.750–0.884) for GRIN1, 0.778 (95% CI: 0.707–0.849) for P2RX1, and 0.842 (95% CI: 0.776–0.908) for P2RY2 (*p* < 0.001 for all). At the optimal cut-off values, GRIN1, P2RX1, and P2RY2 showed sensitivities of 91.4%, 78.5%, and 92.5%, and specificities of 72.00%, 75.3%, and 80.6%, respectively (Table 4) (Figure 1).

## 3. Discussion

FM is a chronic disorder characterized by widespread musculoskeletal pain, somatic symptoms, and increased responsiveness to pain stimuli [16]. Widespread hyperalgesia and/or allodynia result from FM sufferers’ lowered pain threshold. This suggests possible anomalies in the central nervous system’s sensory processing or amplification of pain. Clinical research using functional neuroimaging methods or evaluating changes in neurotransmitter levels that affect pain perception and sensory transmission have confirmed these FM-related findings [17,18,19].

Increased neurotransmitter release causes central sensitization in FM. Substance P and glutamate are two of these neurotransmitters that help activate NMDA receptors, which are in charge of transmitting pain [20]. N-methyl-D-aspartate (NMDA) receptor antagonist therapy has been shown to alleviate pain sensations in a subgroup of FM patients [21]. GRIN1 is an essential subunit for NMDA receptors. Therefore, NMDAR signaling, including GRIN1, has been found to be associated with various neurological diseases [5]. Evidence indicates that NMDA receptor functionality requires the GRIN1 subunit [22]. In this context, GRIN1 appears to contribute to pain-related processes by modulating NMDA receptor-dependent Ca^2+^ signaling, which may enhance pain sensitivity. Consistent with this, increased GRIN1 expression has been observed in pain-related conditions, supporting its potential role in central sensitization mechanisms. Evidence from neuropathic pain models induced by nerve injury indicates that nerve damage is associated with an upregulation of the transcription factor TFAP2A, which in turn leads to increased expression levels of glial GRIN1. These findings support the involvement of GRIN1 in pain-related mechanisms and are consistent with the results of the present study [23]. In an animal model of hyperalgesia, significantly elevated GRIN1 expression in the rostral ventromedial medulla was reported in rats. When the GRIN1 gene in the rostral ventromedial medulla was silenced, pain was found to decrease [24]. In another study, GRIN1 gene expression was suppressed in rats in which formalin-induced pain, particularly central sensitization-related pain, was created. It was observed that GRIN1 reduced NMDA receptor activity, restricted Ca^+2^ influx, and therefore reduced pain [25]. The literature generally provides evidence that elevated GRIN1 levels are associated with pain. Accordingly, high GRIN1 levels were also detected in FM syndrome. This indicates that GRIN1 is a therapeutic target in pain-related diseases such as FM. An increasing body of evidence indicates that extracellular nucleotides play critical roles in regulating neuronal and glial functions in the nervous system through P2 purinergic receptors. Studies have shown that microglia, a type of glial cell regarded as resident macrophages of the central nervous system, express various subtypes of P2X and P2Y receptors, which are critically involved in spinal pain signaling under pathological conditions such as peripheral nerve injury [26].

In a study conducted by D’Amico and colleagues, it was proven that P2X7, a purinergic receptor among the P2X receptors, is associated with FM. According to the study, P2X7 blockade was shown to reduce Caspase-1 activity by suppressing the pyrin domain-containing 3 (NLR3) pathway, decreasing IL-1β and IL-18 levels, and reducing microglial activation, thereby improving central sensitization and consequently FM-related pain [7]. In another study related to FM, the effects of a drug called Suramin on purinergic P2X receptors and NLRP3 inflammasome in the thalamus were investigated, and it was determined whether reserpine-induced FM-like symptoms were reduced. Accordingly, it was noted that P2X4 and P2X7 receptors were overexpressed in the FM-like pain model, that this expression was reduced by Suramin, and that pain symptoms were decreased [8]. While P2X7 and P2X4 receptors have been widely implicated in fibromyalgia-related neuroinflammation, they were not included in the present study due to assay availability and the focus on exploring less-studied circulating purinergic components. Future studies should include a broader panel of P2X receptor subtypes to better delineate their relative contributions. One study reported increased P2RY2 expression following chronic inflammatory pain. It was noted that P2RY2 tends to reorganize itself in high pain situations [27]. In a dorsal root ganglion chronic compression model, increased P2Y_2_ has been shown to be associated with astrocyte activation. There is evidence that pain and inflammation are reduced with the administration of a P2Y_2_ inhibitor (AR-C118925) and pregabalin [15]. Another study reported that several purinergic receptors, including the purinergic P2Y2, were more upregulated in a trigeminal neuralgia model [28].

Although these receptors are predominantly expressed in neuronal and glial cells, accumulating evidence indicates that purinergic and glutamatergic signaling components are also present in peripheral blood cells, including lymphocytes and platelets, particularly under inflammatory conditions, suggesting a potential contribution to circulating protein levels [11,14,17,25].

Purinergic signaling is known to play a significant role in pain-related pathologies. In this study, the role of purinergic receptors in pain-mediating mechanisms was clinically evaluated in FM patients, and significant increases in P2RX1 and P2YR2 protein levels were detected, consistent with previous reports demonstrating increased purinergic receptor activity in chronic pain and inflammatory conditions [7,8,26]. However, the parallel increase in GRIN1 protein levels, which are involved in glutamatergic signaling, suggests that purinergic and glutamatergic pathways are activated together in FM and may contribute to central sensitization processes.

## 4. Materials and Methods

### 4.1. Study Population

This study was conducted on 93 individuals newly diagnosed with FM who presented to the Department of Physical Therapy and Rehabilitation, Sivas Cumhuriyet University Education and Research Hospital, Türkiye. The healthy control group consisted of 93 individuals who presented to the same department and in whom no medical condition was identified. Written informed consent was obtained from all participants prior to inclusion in the study, and the study protocol was approved by the relevant institutional ethics committee in accordance with the Declaration of Helsinki and institutional biosafety regulations (approval for the study was granted by the Sivas Cumhuriyet University Local Ethics Committee, decision no: 2025-10/44). The patient and control groups were randomly selected to be comparable in terms of age and sex. Patients aged 18–65 years who met the 2016 American College of Rheumatology (ACR) diagnostic criteria [29] for fibromyalgia and had chronic widespread pain for at least three months were included in the study. In contrast, individuals with autoimmune, inflammatory, neurodegenerative, or malignant diseases, active infections, pregnancy or breastfeeding, or those who had received corticosteroid, immunosuppressive, or biologic therapies within the previous three months were excluded from the study. In addition, those who received medical treatment within the last month were not included in the study. Patients with menstrual irregularities, perimenopausal symptoms (menstrual cycle irregularities, night sweats, hot flashes, vaginal dryness, etc.), and those who had entered menopause were excluded from the study.

### 4.2. Serum Sample Collection

Fasting blood samples were collected from both patients and healthy individuals and transferred into a single serum separation tube. The samples were centrifuged at 3000 rpm for 10 min at room temperature. The resulting supernatant was carefully transferred into sterile Eppendorf tubes. The samples were stored at −80 °C until analysis to prevent degradation. Confidentiality was strictly maintained: samples were coded with numerical identifiers, and personal information was stored on password-protected computers accessible only to authorized personnel.

### 4.3. ELISA

Serum GRIN1 (Cat. No: E1078Hu, BT LAB, Shanghai, China), P2XR1 (Cat. No: E5824Hu, BT LAB, Shanghai, China), and P2YR2 (Cat. No: E5826Hu, BT LAB, Shanghai, China) protein levels were quantified using enzyme-linked immunosorbent assay (ELISA) kits. The standard curve ranges for GRIN1, P2XR1, and P2YR2 were 70–15000 ng/L, 0.1–40 ng/mL, and 15–3000 ng/L, respectively. The sensitivities of the assays were 35.79 ng/L, 0.051 ng/mL, and 7.24 ng/L, respectively. For all kits, the intra-assay precision was CV < 8%, and the inter-assay precision was CV < 10%. Samples requiring adjustment were diluted to fall within the assay’s dynamic range prior to measurement. All analyses were performed in accordance with the manufacturer’s protocol, and absorbance values were recorded at 450 nm. Low and high standards were included on each plate to ensure the reliability and accuracy of the measurements.

### 4.4. Statistical Analysis

Data obtained from our study were analyzed using IBM SPSS Statistics software (Version 23.0, IBM Corp., Armonk, NY, USA). When the assumptions of parametric tests were met (Kolmogorov–Smirnov test), the significance test was performed when comparing measurements obtained from two independent groups. When the assumptions of parametric tests were not met, the Mann–Whitney U test, logistic regression analysis, odds ratio, and 95% confidence limits were determined. The χ^2^ test was used to evaluate the data obtained by counting. Our data are presented in the tables as arithmetic mean, standard deviation, median, minimum and maximum values, number of individuals, and percentage, with an error level of 0.05.

## 5. Conclusions

In conclusion, this study demonstrates that serum levels of the glutamatergic receptor subunit GRIN1 and purinergic receptors P2RX1 and P2RY2 are significantly higher in patients with FM than in healthy controls. These findings provide clinical evidence supporting the role of both glutamatergic and purinergic signaling pathways in the pathophysiology of FM, particularly in relation to central sensitization and neuroinflammatory processes. As shown by the ROC curve analysis, the observed diagnostic performance of these biomarkers suggests that GRIN1, P2RX1, and P2RY2 can serve as promising circulating markers for the identification of fibromyalgia. In particular, P2RY2 exhibited the highest diagnostic accuracy among the evaluated markers, highlighting its potential importance in disease-related signaling pathways. Importantly, this study contributes to the existing literature by providing the first clinical evidence regarding circulating P2RX1 levels in FM, thus addressing a significant knowledge gap. The simultaneous elevation of glutamatergic and purinergic receptor levels further suggests coordinated activation of these signaling systems, which may play a critical role in amplifying pain signaling and maintaining chronic pain states. From a clinical perspective, these findings support the potential utility of GRIN1, P2RX1, and P2RY2 as non-invasive biomarkers for FM. Furthermore, they may be novel targets for therapeutic interventions aimed at modulating abnormal purinergic and glutamatergic signaling pathways. However, to confirm these findings, clarify the interactions between these receptors, and determine their prognostic and treatment response value in fibromyalgia, further multi-center and longitudinal studies integrating molecular, functional, and clinical parameters are needed.

## 6. Limitations

This study has several limitations that should be acknowledged. First, its cross-sectional design precludes establishing a causal relationship between elevated GRIN1, P2RX1, and P2RY2 levels and the pathophysiology of fibromyalgia. Second, the biomarkers were measured only at the serum protein level using ELISA, without parallel evaluation of gene expression, receptor functionality, or downstream signaling pathways, which limits mechanistic interpretation. Third, although the sample size was adequate for statistical analysis, the study was conducted at a single center and predominantly included female participants, which may limit the generalizability of the findings. Additionally, potential confounding factors such as medication use, pain severity scores, psychological status, and inflammatory markers were not analyzed in relation to receptor levels. Finally, longitudinal follow-up data were not available; therefore, the prognostic value and treatment–response dynamics of these biomarkers could not be assessed. Future multicenter, longitudinal, and mechanistic studies integrating molecular and functional analyses are warranted to validate and extend these findings.

## Figures and Tables

**Table 1:** Comparison of demographic information of individuals in the patient and control groups.

Variable	FM (n = 93)	Control (n = 93)	*p* Values
**Gender**			
Males	2 (2.2%)	1 (1.1%)	0.561
Females	91 (97.8%)	92 (98.9%)	
**Age (year)**			
Means ± SD	44.67 ± 10.44	42.85 ± 6.98	0.164
**Occupation**			
Homemaker	82 (88.2%)	77 (82.8%)	0.245
Nurse	2 (2.2%)	3 (3.2%)	
Student	2 (2.2%)	0 (0.0%)	
Other occupations	7 (7.5%)	13 (14.0%)	
**Sleep disturbance**			
Present	75 (80.6%)	18 (19.4%)	0.001 *
Absent	42 (45.2%)	51 (54.8%)	
**Chronic fatigue**			
Present	91 (97.8%)	72 (77.4%)	0.001 *
Absent	2 (2.2%)	21 (22.6%)	
**Headache**			
Present	78 (83.9%)	53 (57.0%)	0.001 *
Absent	15 (16.1%)	40 (43.0%)	
**Morning fatigue**			
Present	86 (%92.5%)	49 (52.7%)	0.001 *
Absent	7 (7.5%)	44 (47.5%)	
**Dry mouth**			
Present	73 (78.5%)	45 (48.4%)	0.001 *
Absent	20 (21.5%)	48 (51.6%)	
**Lower-extremity numbness**			
Present	72 (77.4%)	42 (45.2%)	0.001 *
Absent	21 (22.6%)	49 (52.7%)	
**Dry eye**			
Present	42 (45.2%)	25 (26.9%)	0.009 *
Absent	51 (54.8%)	68 (73.1%)	
**Difficulty concentrating**			
Present	82 (88.2%)	42 (45.2%)	0.001 *
Absent	11 (11.8%)	51 (54.8%)	
**Perceived soft-tissue swelling**			
Present	59 (63.4%)	41 (44.1%)	0.008 *
Absent	34 (36.6%)	52 (55.9%)	
**Family history of FM**			
Present	33 (35.5%)	0 (0.0%)	0.001 *
Absent	60 (64.5%)	93 (100.0%)	

* Statistically significant *p* < 0.05, Chi-square test with n (%).

**Table 2:** Association between clinical symptoms and FM based on logistic regression analysis.

Clinical Characteristics			95% CI for EXP(B)	
	*p*-Value	Exp(B)	Lower	Upper
Headache	0.023 *	3.388	1.179	9.737
Morning fatigue	0.004 *	4.786	1.628	14.075
Dry mouth	0.050	2.560	1.001	6.551
Lower-extremity numbness	0.005 *	3.801	1.502	9.620
Difficulty concentrating	0.001 *	5.063	1.914	13.397
Perceived soft-tissue swelling	0.042 *	2.454	1.032	5.831

* *p* < 0.05 indicates statistical significance. EXP(B) represents the odds ratio (OR), indicating the likelihood of fibromyalgia associated with each clinical variable.

**Table 3:** Comparison of serum GRIN1, P2RX1, and P2RY2 protein levels between fibromyalgia and controls.

	FM (n = 93)	Control (n = 93)	*p*-Values
**GRIN1** (ng/L)	4759.86 ± 3471.02	3150.72 ± 2736.07	0.001 *^a^
**P2RX1** (ng/mL)	18.73 ± 11.44	13.70 ± 10.13	0.001 *^a^
**P2RY2** (ng/L)	1370.15 ± 985.03	858.95 ± 786.43	0.001 *^a^

^a^ Mann–Whitney U Test, * *p* < 0.05 indicates statistical significance.

**Table 4:** ROC curve analysis of GRIN1, P2RX1, and P2RY2 in FM.

	GRIN1	P2RX1	P2RY2
**AUC (95% CI)**	0.817	0.778	0.842
**Lower limit-Upper limit (95% CI)**	(0.750–0.884)	(0.707–0.849)	(0.776–0.908)
***p*** **-value**	0.001 *	0.001 *	0.001 *
**Cut-off**	2698.33	11.98	781.33
**Sensitivity (%)**	91.4	78.5	92.5
**Specificity (%)**	72.00	75.3	80.6

* *p* < 0.05 indicates statistical significance.

## Data Availability

The original contributions presented in this study are included in the article. Further inquiries can be directed to the corresponding author.

## Abbreviations

The following abbreviations are used in this manuscript:

GRIN1	Glutamatergic
P2RX1	Purinergic Receptor X1
P2RY2	Purinergic Receptor Y1
FM	Fibromyalgia
ELISA	Enzyme-Linked Immunosorbent Assay
ROC	Receiver operating characteristic
AUC	The area under the curve
